# Prosthetic Rehabilitation of a Child Suffering from Hypohidrotic Ectodermal Dysplasia with Complete Anodontia

**DOI:** 10.5005/jp-journals-10005-1155

**Published:** 2012-08-08

**Authors:** Shashi Bala, M Nikhil, Anshul Chugh, Anjali Narwal

**Affiliations:** Professor, Department of Dental Materials, Government Dental College, Rohtak, Haryana, India, e-mail: shashimds@rediffmail.com; Ex-Assistant Professor, Department of Pedodontics, Government Dental College, Rohtak, Haryana, India; Assistant Professor, Department of Prosthodontics, Government Dental College, Rohtak, Haryana, India; Assistant Professor, Department of Oral Pathology, Government Dental College, Rohtak, Haryana, India

**Keywords:** Anodontia, Ectodermal dysplasia, Rehabilitation

## Abstract

A 7-year-old male, described in the case report, exhibited many of the manifestations of ectodermal dysplasia as well as behavioral problems. The treatment to improve his appearance and oral function included a removable prosthesis. The results were significant improvements in speech, masticatory function, and facial esthetics, contributing to the development of normal dietary habits, and the improved and more rapid social integration of the child.

**How to cite this article:** Bala S, Nikhil M, Chugh A, Narwal A. Prosthetic Rehabilitation of a Child Suffering from Hypohidrotic Ectodermal Dysplasia with Complete Anodontia. Int J Clin Pediatr Dent 2012;5(2):148-150.

## INTRODUCTION

Hypohidrotic ectodermal dysplasia (HED) is a hereditary disorder of ectoderm characterized by a congenital dysplasia of one or more ectodermal structures and their accessory appendages. The condition is thought to occur in 1 to 7 per 100,000 live births.^1^ Common manifestations include defective hair follicles and eyebrows, frontal bossing with prominent supraorbital ridges, nasal bridge depression and protuberant lips. Intraorally, common findings are anodontia or hypodontia, conical teeth and consequently, generalized spacing. The patient may suffer from dry skin, hyperthermia, and unexplained high fever as a result of the deficiency of sweat glands.^1-4^ Young children with anodontia caused by hypohidrotic ectodermal dysplasia not only have difficulties in eating and speaking but can also sense that their appearance is different than others. Enabling children with HED to look and act more like their peers through the use of well-fitting and functioning dentures with age-appropriate denture teeth will greatly assist in their transitioning into the school years. Although denture fabrication requires multiple patient appointments and good cooperation, it is shown that even young children can cooperate for the denture-making process. The desire to be like others who have teeth can be a motivator for cooperation in even the young child.

## CASE REPORT

**Fig. 1:**
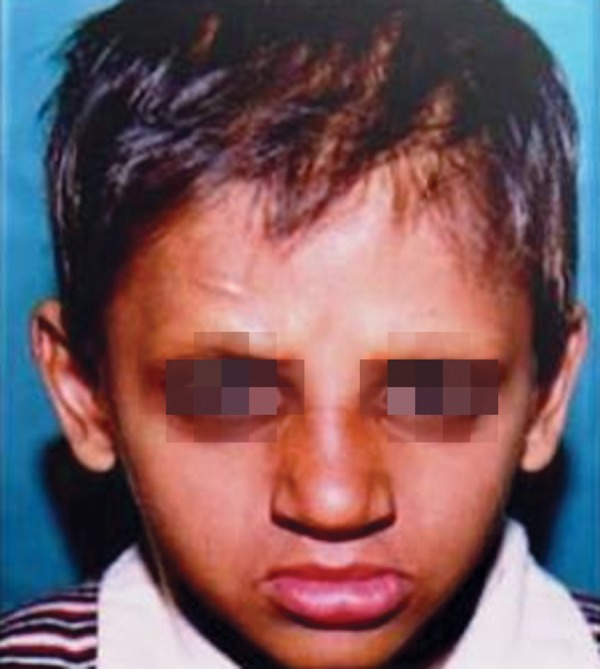
Pretreatment facial profile of the child

**Fig. 2:**
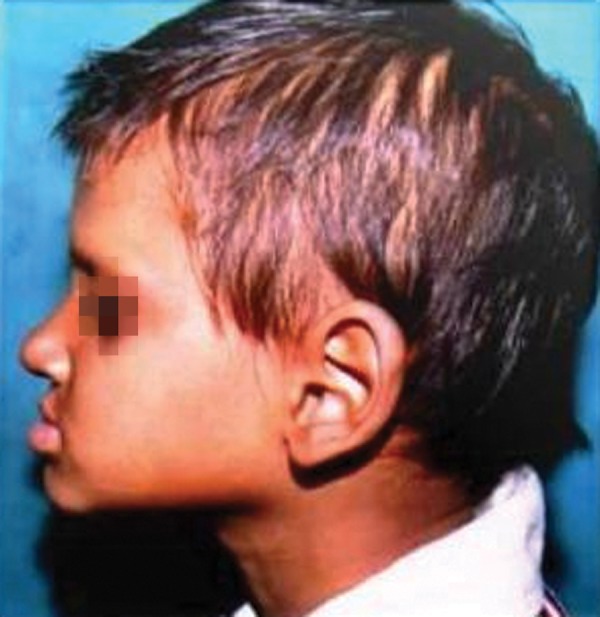
Lateral profile, protuberant lips

A 7-year-old boy reported to the Government Dental College with the complaint of missing teeth, inability to eat and difficulty in speech. Family and medical history was non contributory. He exhibited classical features of ectodermal dysplasia; anodontia, hypohidrosis, scanty eyebrows and eyelashes, saddle nose, diminished lower facial height, protuberant lips (Figs 1 and 2).

**Fig. 3:**
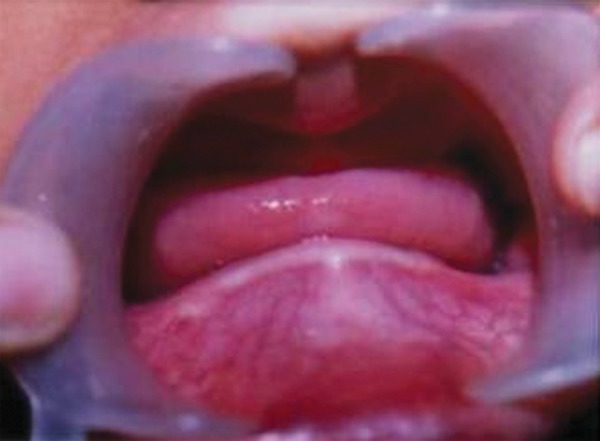
Edentulous ridges

**Fig. 4:**
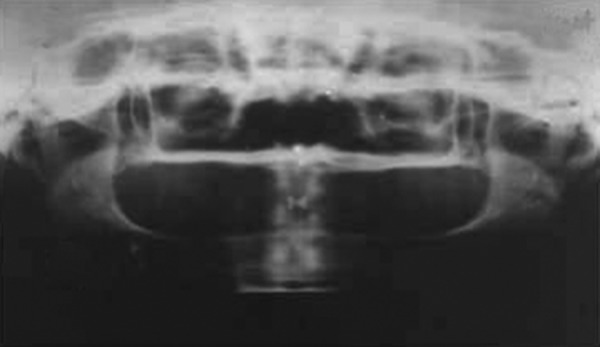
Radiograph showing complete anodontia

Intraoral examination revealed missing deciduous and permanent teeth which was confirmed by orthopantomograph with no tooth buds seen (Figs 3 and 4). Edentulous ridges were both deficient in height and width. The oral mucosa was slightly dry with enlarged tongue.

Complete dentures were planned keeping in mind the requirement of esthetics, mastication, speech and overall psychological development.

**Fig. 5:**
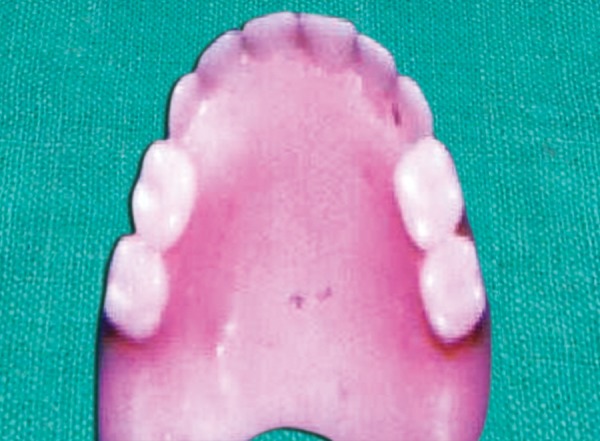
Maxillary complete denture

**Fig. 6:**
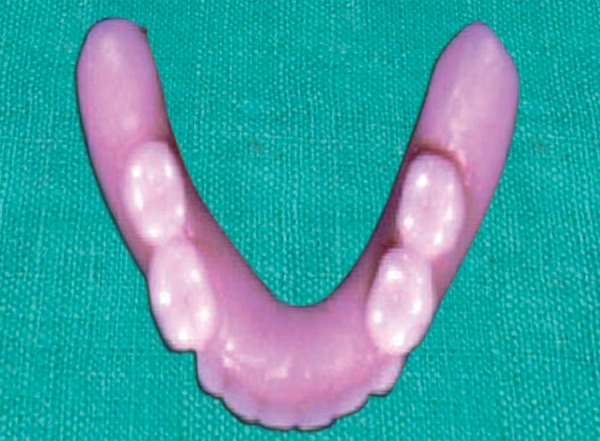
Mandibular complete denture

**Fig. 7:**
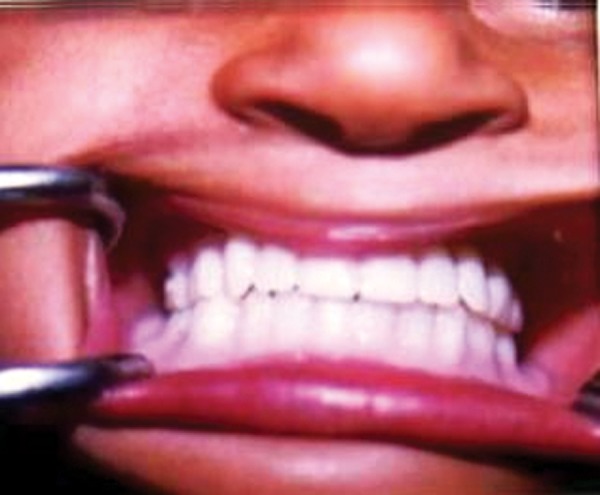
Denture inserted

**Fig. 8:**
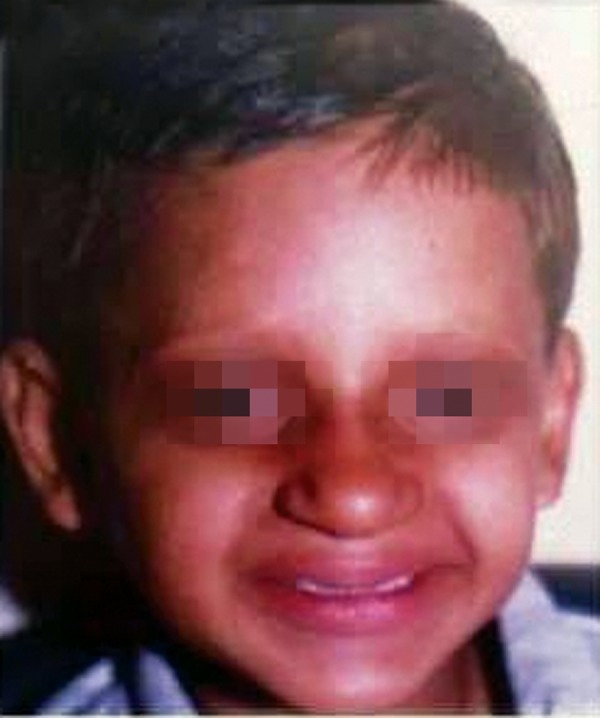
Posttreatment facial profile of the child

**Fig. 9:**
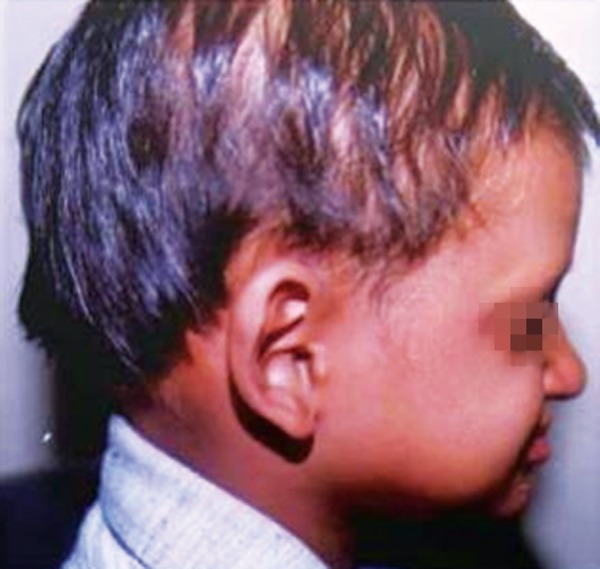
Posttreatment lateral profile, esthetics achieved

- Diagnostic impressions were created using irreversible hydrocolloid impression material employing the smallest stock tray. Custom trays were fabricated in auto- polymerizing resin.
- Heat-processed bases were fabricated on the master casts. Occlusal rims were fabricated chair side, and the appropriate occlusal vertical dimension (OVD) was determined. A fox plane was used to confirm the occlusal plane, and the centric jaw relation was recorded using a silicone-based bite registration material.
- Pediatric mold denture teeth were chosen to simulate the natural dentition of a 7-year-old child. The trial bases were tried in the patient's mouth. Esthetics, OVD and centric relation records were confirmed. The patient and his mother evaluated and approved the teeth setup.
- The dentures were fabricated in heat-processed acrylic resin, finished, polished, inserted and pressure spots were checked and adjusted using pressure-indicating paste (Figs 5 to 7).
- Postinsertion instructions were given to the patient and parents. Retention of maxillary denture was good and patient slowly adapted to the maxillary denture. Patients self-esteem improved as he started socializing.
- Patients esthetics improved remarkably and he was very happy (Figs 8 and 9).
- Further treatment will include modifications of the dentures by relining or replacement as per need of skeletal growth.

## DISCUSSION

Early prosthetic treatment in children with HED is important. It has been reported that child's self image is complete by 4 to 5 years of age. Therefore cosmetic and prosthodontic measures should be instituted as early as possible to have the child resemble his peers.^4^ Prosthetic intervention can be done with a child as young as 2 or 3 years if the child is cooperative (Hickey, 2001).^2^ This also allows the child to adjust with the prosthesis or appliance and develop normal appearance, speech, mastication and swallowing as well as temporo- mandibular joint function. Apart from dental benefits, an early age intervention also provides psychosocial benefits. The unesthetic appearance, poor self image, school/job related discrimination often accompanies ectodermal dysplasia syndrome which has a negative psychological effect on the patient. Thus, management of the orofacial disfigurement provides the patient with some measure of confidence. Treatment generally includes a removable and/or fixed partial denture, an overdenture, complete denture prosthesis or an implant retained prosthesis.^5^

The treatment option preferred in our case was of a removable partial denture considering his present age.

Although complete dentures are a simple, inexpensive and reversible prosthodontic option, parents of these patients should be educated about the future possibilities for dental implant placement, with the eventual goal of obtaining an implant- supported prosthesis. The replacement of teeth by implants is usually restricted to patients with completed craniofacial growth. Implant insertions in children or adolescents are circumvented due to several unfavorable potential effects including trauma to tooth germs, tooth eruption disorders and multidimensional restrictions of skeletal craniofacial growth. The literature is clear about the long-term success of dental implants in children.^5,6^ Various implant-based options shown to be successful in fully grown adult edentulous patients with ED range from over dentures,^5-7^ to complete fixed prostheses,^2,5,7^ to complete fixed prostheses using zygomatic implants.^8,9^

### Why the Paper is Important to Pediatric Dentist?

- Children should be given every opportunity to develop to their fullest potential.
- Early diagnosis and treatment is vital in restoring esthetics, speech and mastication.
- The dentist can make a significant contribution to the overall development and well being of a child with HED.
- There is a lack of evidence of one technique being superior; thus, a simplified approach is necessary. This will perhaps encourage more dentists to treat these patients.

### What this Paper Adds?

- Considering the importance of early prosthodontic manangement it is important to instill awareness among the parents regarding early management.
- Psychotherapy or counseling may be helpful to the entire family in the management and behavioral adjustment of the child.
- The principles described in this article can assist the clinician in using this simple therapeutic option to provide esthetic, functional and psychological benefits for children and thus contribute to their overall development and well being.
